# Determination of cognitive domain involvement in a sample of patients diagnosed with schizophrenia and cardiovascular risk factors

**DOI:** 10.1192/j.eurpsy.2021.1440

**Published:** 2021-08-13

**Authors:** J. García Eslava, A. Marzán Gutierrez, N. Riera Molist, S. Escoté Llobet, Q. Foguet-Boreu

**Affiliations:** 1 Mental Health Department, Consorci Hositalari Vic, Vic, Spain; 2 Mental Health Department, Consorci Hospitalari de Vic, Vic, Spain

**Keywords:** schizophrénia, cognitive impairment, cognitive domains, cardiovascular risk

## Abstract

**Introduction:**

Schizophrenia it’s a deteriorating illness, where the cognitive impairment it’s one of the predominant components in this process. Theory of neurodevelopment, the most widely recognized, explains that cognition will depend most of it, on premorbid development. However, other factors explain this impairment, such as the cardiovascular risk factors (CVRF).

**Objectives:**

The purpose of this study is to determine cognitive impairment and the domains affected in a sample of patients who suffered schizophrenia and almost one CVRF.

**Methods:**

Cross-sectional study. Patients diagnosed with schizophrenia and at least one poorly controlled CVRF (diabetes, hypercholesterolemia, arterial hypertension or active smoking) were selected. Screen for Cognitive Impairment in Psychiatry (SCIP) scale was used to evaluate cognitive impairment and the domains affected.

**Results:**

Preliminary data of twenty patients were included (60% men, mean age: 50 years). At CVRF in the sample, no diabetes was found, 75% had hypercholesterolemia, 15% arterial hypertension and 20% active smoking. SCIP scale showed deficits in word learning and delayed learning in 95% of the sample (n=19). The domain less affected was verbal fluency, affected in 55% of the sample (n=11). Additionally, moderate to severe cognitive impairment was observed in 65% of the sample (n=13).

**Conclusions:**

More than half of the patients with schizophrenia and CVRF have a moderate to severe cognitive impairment. Intervention at CVRF could reduce the severity of cognitive impairment, improving functionality in these patients.

